# High-sensitivity c-reactive protein and gamma-glutamyl transferase levels are synergistically associated with metabolic syndrome in community-dwelling persons

**DOI:** 10.1186/1475-2840-9-87

**Published:** 2010-12-09

**Authors:** Ryuichi Kawamoto, Yasuharu Tabara, Katsuhiko Kohara, Tetsuro Miki, Tomo Kusunoki, Shuzo Takayama, Masanori Abe, Tateaki Katoh, Nobuyuki Ohtsuka

**Affiliations:** 1 Department of Community Medicine, Ehime University Graduate School of Medicine; Ehime 791-0295, Japan; 2 Geriatric Medicine, Ehime University Graduate School of Medicine; Ehime 791-0295, Japan; 3 Department of Internal Medicine, Seiyo Municipal Nomura Hospital, Ehime 797-1212, Japan

## Abstract

**Background:**

Metabolic syndrome (MetS) is associated with an increased risk of major cardiovascular events. Increased high-sensitivity C-reactive protein (hsCRP) levels are associated with MetS and its components. Changes in gamma-glutamyl transferase (GGT) levels in response to oxidative stress are also associated with MetS, and the levels could be modulated by hsCRP.

**Methods:**

From a single community, we recruited 822 men (mean age, 61 ± 14 years) and 1,097 women (63 ± 12 years) during their annual health examination. We investigated whether increased hsCRP and GGT levels are synergistically associated with MetS and insulin resistance evaluated by Homeostasis of model assessment of insulin resistance (HOMA-IR).

**Results:**

Of these subjects, 141 men (17.2%) and 170 women (15.5%) had MetS. Participants with MetS had a higher hsCRP and GGT level than those without MetS in both genders, and the HOMA-IR increased significantly in correlation with an increase in hsCRP and GGT. In men, the adjusted odds ratios (95% confidence interval) for MetS across tertiles of hsCRP and GGT were 1.00, 1.69 (1.01-2.80), and 2.13 (1.29-3.52), and 1.00, 3.26 (1.84-5.78) and 6.11 (3.30-11.3), respectively. In women, the respective corresponding values were 1.00, 1.54 (0.92-2.60), and 3.08 (1.88-5.06), and 1.00, 1.70 (1.04-2.79) and 2.67 (1.66-4.30). The interaction between increased hsCRP and GGT was a significant and independent determinant for MetS and insulin resistance in both genders.

**Conclusions:**

These results suggested that higher CRP and GGT levels were synergistically associated with MetS and insulin resistance, independently of other confounding factor in the general population.

## Introduction

Metabolic syndrome (MetS), a clustering of cardiovascular risk factors such as insulin resistance, hypertension, glucose intolerance, hypertriglyceridemia, and low high-density lipoprotein cholesterol (HDL-C) levels, is a major worldwide public health problem. MetS increases the risk of atherosclerotic disease, diabetes [1,2], and cardiovascular disease (CVD) [3]. MetS affects 13.3% to 24.4% of Japanese men ≥ 30 years of age [4,5]. With the continuous increase in obesity prevalence in Japan, MetS may become even more common.

Recent data support the concept that high-sensitivity C-reactive protein (hsCRP) is an inflammatory marker and independent predictor reflecting the early stage of CVD [6]. Several studies have demonstrated that hsCRP is induced by cytokines produced by accumulated adipocytes, and then increases in subjects with MetS [7,8].

Serum Gamma-Glutamyl Transferase is an enzyme present on cell surfaces and in serum that contributes to the extracellular catabolism of glutathione (GSH), but most serum GGT is derived from the liver [9]. Gamma-Glutamyl Transferase (GGT) is also a clinical marker of several factors: alcohol consumption, body fat content [10], plasma lipid/lipoproteins [11,12] and glucose levels [12-14], blood pressure [12,14], and metabolic syndrome [14,15]. It is also associated with CVD [14,15] and CVD mortality [14-16]. In addition, Taki et al. [17] reported that GGT showed a significant correlation with hsCRP, suggesting a possible interaction between these two key makers. However, there are few reports on the relationship between CRP, GGT and MetS in Japan.

The aim of this study was to determine whether increased hsCRP and GGT levels are interactively associated with MetS, and we examined cross-sectional data from Japanese community-dwelling participants.

## Methods

### Subjects

Participants were recruited at the time of their annual health examination in a rural town located in Ehime prefecture, Japan. Participants were recruited at the time of their annual health examination in a rural town with a total population of 11,136 (as of April 2002) and located in Ehime prefecture, Japan, in 2002. Among the 9,133 adults (4,395 male) aged 19 to 90 years in this population, a random sample of 3,164 (34.6%) subjects was recruited. Other characteristics such as smoking and alcohol habits, and medication, were investigated by individual interviews that were conducted using a structured questionnaire. The final study sample included 1,919 eligible persons. All procedures were approved by the Ethics Committee of Ehime University School of Medicine and each subject gave informed consent to participate.

### Evaluation of Risk Factors

Information on demographic characteristics and risk factors was collected using the clinical files. Body mass index was calculated by dividing weight (in kilograms) by the square of the height (in meters). We measured blood pressure with an appropriate-sized cuff on the right upper arm of the subjects in a sedentary position using an automatic oscillometric blood pressure recorder (BP-103i; Colin, Aichi, Japan) while they were seated after having rested for at least 5 min. Smoking status was defined as the number of cigarette packs per day multiplied by the number of years smoked (pack year), and the participants were classified into never smokers, past smokers, light smokers (<30 pack year) and heavy smokers (≥30 pack year). The daily alcohol consumption was measured using the Japanese liquor unit in which a unit corresponds to 22.9 g of ethanol, and the participants were classified into never drinkers, occasional drinkers (<1 unit/day), light drinkers (1-1.9 unit/day), and heavy drinkers (≥2 unit/day). Total cholesterol (T-C), triglycerides (TG), HDL-C, fasting blood glucose (FBG), creatinine (enzymatic method), uric acid, immuno-reactive insulin (IRI), plasma high molecular weight (HMW) adiponectin (FUJIREBIO, Tokyo, Japan), hsCRP, and GGT were measured during fasting. Plasma hsCRP concentration was measured using a Behring BN II nephelometer (Dade Behring Inc., Marburg, Germany) and this inter- and intraassay coefficient variations was 3.2 and 6.7%, respectively. Serum GGT concentration was assayed with an automatic analyzer (TBA-c6000, TOSHIBA, Tokyo) and this intraassay-coefficient of variation was 0.87 to 2.11%. Low-density lipoprotein cholesterol (LDL-C) level was calculated by the Friedewald formula. Participants with TG levels ≥400 mg/dL were excluded. Estimated GFR was calculated using the following equation: eGFR = 194×Cr^-1.094^×Age^-0.287^×0.739 (if female) [18]. Participants with an eGFR <30 mL/min/1.73 m^2 ^were excluded. Homeostasis of model assessment of insulin resistance (HOMA-IR) was calculated from FBG and IRI levels using the following formula: {FBG (mg/dL) × IRI (mU/mL)}/405 [19]. Insulin resistance was defined as a HOMA-IR ≥2.6.

### Metabolic Syndrome

We applied condition-specific cutoff points for MetS based on the modified criteria of the National Cholesterol Education Program's Adult Treatment Panel (NCEP-ATP) III report [20]. Metabolic syndrome was defined as subjects with at least three or more of the following five conditions: 1) obesity: BMI ≥25.0 kg/m^2 ^according to the guidelines of the Japanese Society for the Study of Obesity (waist circumference was not available in this study) [21,22]; 2) raised BP with systolic blood pressure (SBP) ≥130 mmHg and/or diastolic blood pressure (DBP) ≥85 mmHg, and/or current treatment for hypertension; 3) Hypertriglyceridemia with a TG level ≥1.69 mmol/L (150 mg/dL); 4) low HDL cholesterolemia with a HDL-C <1.04 mmol/L (40 mg/dL) in men and 1.30 mmol/L (50 mg/dL) in women; and 5) IFG with a FBG level ≥6.1 mmol/L (110 mg/dL) or current treatment for diabetes mellitus.

### Statistical Analysis

Data are presented as the mean ± standard deviation (SD) unless otherwise specified, and in the cases of parameters with non-normal distributions (TG, IRI, FBG, HOMA-IR, and GGT) the data are shown as median (interquartile range) values. In all analyses, parameters with non-normal distributions were used after log-transformation. As several background differences between men and women were demonstrated by previous studies [2,16,22], statistical analysis was performed according to sex using PASW Statistics 17.0 (Statistical Package for Social Science Japan, Inc., Tokyo, Japan). The differences among groups categorized by sex and presence of MetS were analyzed by Student's t-test for continuous variables or the χ^2 ^-test for categorical variables. Correlations between various characteristics and HOMA-IR were determined using Pearson's correlation. Subjects were divided into three groups based on tertiles of serum GGT and hsCRP within sex and then combined to avoid the gender differences. Multiple logistic regression analysis was used to evaluate the contribution of confounding factors for MetS and each component of MetS. The synergistic effect of CRP and GGT was evaluated using a general linear model adjusted for the following parameters: age, smoking status, alcohol consumption, uric acid, and estimated glomerular filtration rate. In addition, we demonstrated whether the ORs for MetS and insulin resistance dose-dependently increased in relation to hsCRP and GGT in subgroups of confounding factors which effected on MetS and insulin resistance (e.g., age, alcohol consumption, uric acid, medication, HMW adiponectin). A *p*-value < 0.05 was considered significant.

## Results

### Characteristics of subjects

The characteristics of the study participants in relation to sex are illustrated in Table 1. The study included 822 men, aged 61 ± 14 (range, 20-89) years, and 1,097 women, aged 63 ± 12 (range, 21-88) years. Smoking status, alcohol consumption, history of CVD, DBP, TG, uric acid, FBG, hsCRP, and GGT were higher in men than in women, but age, HDL-C, LDL-C, presence of antilipidemic medication, IRI, HOMA-IR, and HMW adiponectin were higher in women than in men. There was no inter-group difference in BMI, SBP, presence of antihypertensive medication, eGFR, and diabetic medication.

**Table 1:** Characteristics of subjects categorized according to sex

Characteristics	Men (N = 822)	Women (N = 1,097)	*P*-value*
Age (years)	61 ± 14	63 ± 12	<0.001
Body mass index (kg/m^2^)	23.6 ± 3.0	23.4 ± 3.4	0.154
Smoking status, %	41.6/24.8/14.6/19.0	97.5/0.8/1.5/0.1	<0.001
Alcohol consumption, %	14.6/28.5/34.3/22.6	64.6/30.0/4.9/0.5	<0.001
History of cardiovascular disease, %	9.9	6.8	0.011
Systolic blood pressure (mmHg)	140 ± 20	139 ± 23	0.064
Diastolic blood pressure (mmHg)	84 ± 11	80 ± 12	<0.001
Antihypertensive medication, %	24.7	25.6	0.343
Triglycerides (mmol/L)	1.08 (0.81-1.55)	1.01 (0.75-1.39)	<0.001
HDL cholesterol (mmol/L)	1.53 ± 0.38	1.68 ± 0.40	<0.001
LDL cholesterol (mmol/L)	2.86 ± 0.81	3.26 ± 0.76	<0.001
Antilipidemic medication, %	3.9	6.7	0.005
Serum uric acid (μmol/L)	351 ± 83	265 ± 62	<0.001
eGFR (mL/min/1.73 m^2^)	81.1 ± 17.0	80.2 ± 17.0	0.212
Immuno-reactive insulin (mU/mL)	4.7 (3.1-7.3)	6.0 (4.1-8.7)	<0.001
Fasting blood glucose (mmol/L)	5.27 (4.94-5.77)	5.11 (4.83-5.55)	<0.001
HOMA-IR	1.12 (0.70-1.86)	1.39 (0.91-2.08)	<0.001
Diabetic medication, %	4.0	3.2	0.200
HMW adiponectin (μg/mL)	3.40 (2.01-5.34)	6.68 (4.39-9.65)	<0.001
hsCRP (mg/L)	0.520 (0.280-1.073)	0.430 (0.220-0.870)	<0.001
GGT (IU/L)	38 (25-68)	20 (16-28)	<0.001

Data presented are mean ± standard deviation. HDL, high-density lipoprotein; LDL, low-density lipoprotein; eGFR, estimated glomerular filtration rate; HMW, high molecular weight; hsCRP, high sensitivity C-reactive protein; GGT, gamma-glutamyl transferase. Data for triglycerides, fasting blood glucose, HOMA-IR, HMW adiponectin, hsCRP, and GGT were skewed and are presented as median (interquartile range) values, and were log-transformed for analysis. *P-value from Student's t-test for continuous variables or from χ^2^-test for categorical variables.

### Association between various characteristics, and MetS and insulin resistance

Table 2 shows the risk of MetS and abnormalities of its components in relation to hsCRP and GGT among 822 men and 1,097 women. Of these, 141 men (17.2%) and 170 women (15.5%) had MetS. As shown Table 2, BMI, SBP, DBP, TG, LDL-C, FBG, HOMA-IR, presence of diabetic medication, hsCRP, and GGT showed a higher level in participants with MetS than those without in both genders, but HDL-C and HMW adiponectin showed a lower level in those with MetS. Age, presence of antilipidemic medication, and uric acid were higher only in women with MetS, but eGFR was lower in women with MetS. In men, the HOMA-IR increased significantly in correlation with an increase in BMI, SBP, DBP, presence of antihypertensive medication, TG, LDL-C, presence of antilipidemic medication, uric acid, FBG, presence of diabetic medication, hsCRP, and GGT, but decrease in age, HDL-C, and HMW adiponectin. In women, the HOMA-IR increased significantly in correlation with an increase in age, BMI, SBP, DBP, presence of antihypertensive medication, TG, LDL-C, presence of antilipidemic medication, uric acid, FBG, presence of diabetic medication, hsCRP, and GGT, but decrease in HDL-C, eGFR, and HMW adiponectin.

**Table 2:** Association between various characteristics, and metabolic syndrome components and Insulin resistance

	Metabolic syndrome						HOMA-IR	
		
	Men, N = 822			Women, N = 1,097			Men, N = 822	Women, N = 1,097
							Pearson's correlation	
Characteristics	No N = 681	Yes N = 141	*P *-value*	No N = 927	Yes N = 170	*P*-value*	*r (P-value)*	*r (P-value)*
Age (years)	61 ± 14	60 ± 12	0.342	62 ± 12	66 ± 9	0.001	-0.141 (<0.001)	0.100 (0.001)
Body mass index (kg/m^2^)	23.1 ± 2.8	26.1 ± 2.6	<0.001	22.8 ± 2.9	26.8 ± 3.6	<0.001	0.547 (<0.001)	0.516 (<0.001)
Smoking status, %	41.4/23.9/15.7/18.9	42.6/29.1/9.2/19.1	0.199	97.4/0.8/1.7/0.1	98.2/1.2/0.6/0	0.636	-0.081 (0.020)	0.019 (0.536)
Alcohol consumption, %	14.4/28.0/35.4/22.2	15.6/30.5/29.1/24.8	0.555	63.3/30.9/5.4/0.4	71.8/25.3/2.4/0.6	0.120	-0.064 (0.066)	-0.049 (0.104)
History of cardiovascular disease, %	23.6	29.8	0.078	6.3	10.0	0.058	0.048 (0.171)	0.051 (0.090)
Systolic blood pressure (mmHg)	139 ± 20	156 ± 17	0.001	136 ± 23	150 ± 20	<0.001	0.137 (<0.001)	0.245 (<0.001)
Diastolic blood pressure (mmHg)	84 ± 11	88 ± 10	<0.001	79 ± 12	86 ± 10	<0.001	0.181 (<0.001)	0.230 (<0.001)
Antihypertensive medication, %	23.6	29.8	0.078	21.1	50.0	<0.001	0.124 (<0.001)	0.195 (<0.001)
Triglycerides (mmol/L)	1.00 (0.77-1.33)	1.89 (1.52-2.60)	<0.001	0.94 (0.72-1.23)	1.76 (1.21-2.12)	<0.001	0.392 (<0.001)	0.365 (<0.001)
HDL cholesterol (mmol/L)	1.58 ± 0.37	1.27 ± 0.36	<0.001	1.74 ± 0.38	1.32 ± 0.32	<0.001	-0.319 (<0.001)	-0.232 (<0.001)
LDL cholesterol (mmol/L)	2.83 ± 0.79	3.01 ± 0.92	0.019	3.24 ± 0.74	3.40 ± 0.85	0.011	0.206 (<0.001)	0.200 (<0.001)
Antilipidemic medication, %	3.7	5.0	0.301	5.7	11.8	0.005	0.070 (0.044)	0.088 (0.004)
Serum uric acid (μmol/L)	349 ± 81	360 ± 91	0.146	261 ± 61	291 ± 58	<0.001	0.171 (<0.001)	0.252 (<0.001)
eGFR (mL/min/1.73 m^2^)	80.9 ± 16.7	82.3 ± 18.6	0.385	80.9 ± 17.1	76.3 ± 16.2	0.001	-0.009 (0.791)	-0.068 (0.024)
Fasting blood glucose (mmol/L)	5.22 (4.94-5.61)	6.11 (5.27-6.83)	<0.001	5.05 (4.83-5.44)	5.61 (5.11-6.61)	<0.001	0.474 (<0.001)	0.540 (<0.001)
HOMA-IR	1.01 (0.65-1.64)	2.21 (1.43-3.20)	<0.001	1.27 (0.85-1.79)	2.52 (1.80-3.79)	<0.001	----------	----------
Diabetic medication, %	2.8	9.9	<0.001	2.2	8.8	<0.001	0.112 (0.001)	0.133 (<0.001)
HMW adiponectin (μg/mL)	3.63 (2.18-5.65)	2.28 (1.60-3.69)	<0.001	7.09 (4.69-10.3)	4.48 (3.12-7.17)	<0.001	-0.331 (<0.001)	-0.340 (<0.001)
hsCRP (mg/L)	0.480 (0.275-1.015)	0.690 (0.400-1.535)	<0.001	0.380 (0.200-0.760)	0.750 (0.390-1.613)	<0.001	0.163 (<0.001)	0.290 (<0.001)
GGT (IU/L)	35 (24-61)	55 (36-102)	<0.001	20 (15-26)	25 (19-35)	<0.001	0.240 (<0.001)	0.328 (<0.001)

Data presented are mean ± standard deviation. Metabolic syndrome was defined as having three or more of the following conditions: obesity, raised blood pressure, hypertriglyceridemia, low HDL- cholesterolemia, and impaired fasting blood glucose. Data for triglycerides, fasting blood glucose, HOMA-IR, HMW adiponectin, hsCRP, and GGT were skewed and are presented as median (interquartile range) values, and were log-transformed for analysis. *P-value from Student's t-test for continuous variables or from χ^2^-test for categorical variables.

### The adjusted odds ratio for MetS. its components, and insulin resistance in relation to tertiles of hsCRP and GGT

As shown Table 3, after adjustments for age, smoking status, alcohol consumption, uric acid, and eGFR, the prevalence rate of MetS increased significantly in relation to hsCRP and GGT in both genders. In men, the ORs (95% CI) for MetS across tertiles of hsCRP and GGT were 1.00, 1.69 (1.01-2.80), and 2.13 (1.29-3.52), and 1.00, 3.26 (1.84-5.78) and 6.11 (3.30-11.3), respectively. In women, the ORs (95% CI) for MetS across tertiles of hsCRP and GGT were 1.00, 1.54 (0.92-2.60), and 3.08 (1.88-5.06), and 1.00, 1.70 (1.04-2.79) and 2.67 (1.66-4.30), respectively. In men, the ORs of hsCRP were significantly high for MetS components of obesity, low HDL cholesterolemia, and impaired fasting glucose, and the ORs of GGT were significantly high for obesity, raised blood pressure, hypertriglyceridemia, and impaired fasting glucose. In women, the ORs of hsCRP were significantly high for MetS components of obesity, hypertriglyceridemia, low HDL cholesterolemia, and impaired fasting glucose, and the ORs of GGT were significantly high for obesity, hypertriglyceridemia, and impaired fasting glucose. The ORs for HOMA-IR ≥2.6 also increased significantly in relation to hsCRP and GGT in both genders.

**Table 3:** The adjusted odds ratios for metabolic syndrome, its components, and insulin resistance in relation to tertiles of hsCRP and GGT

	Men, N = 822						Women, N = 1,097					
		
	Tertile of hsCRP			Tertile of GGT			Tertile of hsCRP			Tertile of GGT		
				
	1st	2nd	3rd	1st	2nd	3rd	1st	2nd	3rd	1st	2nd	3rd
Characteristics	<0.360	0.360-0.800	>0.800 mg/L	<29	29-54	>54 IU/L	<0.280	0.280-0.650	>0.650 mg/L	<18	18-24	>24 IU/L
Metabolic syndrome	29 (20.6%)	49 (34.8%)	63 (44.7%)	19 (13.5%)	51 (36.2%)	71 (50.4%)	25 (14.7%)	47 (27.6%)	98 (57.6%)	29 (17.1%)	54 (31.8%)	87 (51.2%)
OR (95% CI)	1.00	1.69 (1.01-2.80)	2.13 (1.29-3.52)	1.00	3.26 (1.84-5.78)	6.11 (3.30-11.3)	1.00	1.54 (0.92-2.60)	3.08 (1.88-5.06)	1.00	1.70 (1.04-2.79)	2.67 (1.66-4.30)
Obesity	61 (24.9%)	72 (29.4%)	112 (45.7%)	40 (16.3%)	88 (35.9%)	117 (47.8%)	49 (15.1%)	103 (31.7%)	173 (53.2%)	66 (20.3%)	107 (32.9%)	152 (46.8%)
OR (95% CI)	1.00	1.16 (0.76-1.76)	2.21 (1.47-3.32)	1.00	2.65 (1.71-4.12)	4.75 (2.92-7.72)	1.00	2.15 (1.46-3.18)	4.04 (2.74-5.97)	1.00	1.60 (1.11-2.32)	2.17 (1.50-3.12)
Raised blood pressure	186 (31.2%)	201 (33.7%)	209 (35.1%)	184 (30.9%)	208 (34.9%)	204 (34.2%)	220 (30.0%)	249 (34.0%)	264 (36.0%)	236 (32.2%)	245 (33.4%)	252 (34.4%)
OR (95% CI)	1.00	1.34 (0.88-2.03)	1.20 (0.78-1.85)	1.00	2.13 (1.38-3.29)	2.72 (1.67-4.43)	1.00	1.02 (0.71-1.46)	0.97 (0.66-1.42)	1.00	1.22 (0.85-1.74)	1.19 (0.82-1.73)
Hypertriglyceridemia	49 (27.7%)	62 (35.0%)	66 (37.3%)	28 (15.8%)	51 (28.8%)	98 (55.4%)	31 (19.4%)	49 (30.6%)	80 (50.0%)	26 (16.3%)	40 (25.0%)	94 (58.8%)
OR (95% CI)	1.00	1.21 (0.77-1.88)	1.21 (0.77-1.88)	1.00	2.03 (1.22-3.37)	5.43 (3.18-9.26)	1.00	1.20 (0.73-1.97)	1.68 (1.03-2.71)	1.00	1.47 (0.87-2.49)	3.92 (2.40-6.38)
Low HDL cholesterolemia	10 (15.9%)	26 (41.3%)	27 (42.9%)	23 (33.8)	24 (38.1%)	16 (25.4%)	42 (23.7%)	55 (31.1%)	80 (45.2%)	53 (29.9%)	59 (33.3%)	65 (36.7%)
OR (95% CI)	1.00	2.85 (1.33-6.12)	2.79 (1.28-6.07)	1.00	0.94 (0.50-1.76)	0.76 (0.36-1.64)	1.00	1.29 (0.83-2.00)	1.95 (1.25-3.02)	1.00	1.12 (0.74-1.69)	1.17 (0.77-1.79)
Impaired fasting glucose	46 (23.8%)	70 (36.3%)	77 (39.9%)	50 (25.9%)	62 (32.1%)	81 (42.0%)	28 (19.3%)	38 (26.2%)	79 (54.5%)	28 (19.3%)	46 (31.7%)	71 (49.0%)
OR (95% CI)	1.00	1.73 (1.12-2.67)	1.82 (1.17-2.82)	1.00	1.54 (0.99-2.40)	2.81 (1.72-4.59)	1.00	1.12 (0.66-1.90)	2.24 (1.37-3.66)	1.00	1.55 (0.93-2.59)	2.36 (1.44-3.88)
HOMA-IR ≥2.6†	12 (11.9%)	37 (36.6%)	52 (51.5%)	18 (17.8%)	36 (35.6%)	47 (46.5%)	23 (13.3%)	49 (28.3%)	101 (58.4%)	18 (10.4%)	58 (33.5%)	97 (56.1)
OR (95% CI)	1.00	3.40 (1.08-3.73)	5.14 (2.60-10.1)	1.00	2.01 (1.08-3.73)	3.00 (1.55-5.82)	1.00	1.78 (1.04-3.04)	3.51 (2.11-5.86)	1.00	3.13 (1.78-5.49)	4.99 (2.89-8.64)

Study subjects were divided into three groups (tertiles) according to hsCRP and GGT levels within sex. OR, odds ratio; CI, confidence interval.

† Insulin resistance was defined as HOMA-IR ≥2.6. Adjusted for age, smoking status, alcohol consumption, uric acid, and estimated glomerular filtration rate.

### Synergistic effect of GGT and hsCRP on mean accumulating number of MetS components and insulin resistance

**Figure 1:**
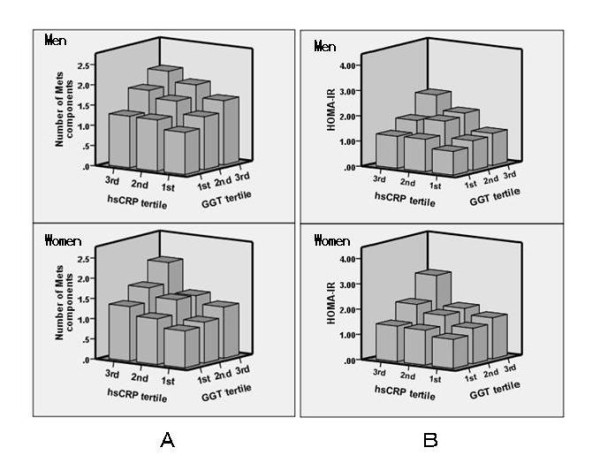
**Synergistic effect of GGT and hsCRP**. A, Mean accumulating number of metabolic syndrome components: obesity, raised blood pressure, hypertriglyceridemia, low high-density lipoprotein cholesterolemia, and impaired fasting glucose. B, HOMA-IR. Study subjects were divided into three groups (tertiles) according to GGT and hsCRP levels. Each tertile was calculated within sex and then combined to avoid the gender differences.

In addiction to their direct associations, we observed a synergistic effect between hsCRP and GGT (Figure 1). In Figure 1 subjects were divided into three groups (tertiles) according to CRP and GGT levels within sex. We assessed the statistical significance of the synergistic relationship using a general linear model with the following confounding factors: age, smoking status, alcohol consumption, uric acid, eGFR (Table 4). The interaction between increased hsCRP and GGT was a significant and independent determinant for MetS and HOMA-IR ≥2.6 in both genders.

**Table 4:** Interaction between hsCRP and GGT for metabolic syndrome and insulin resistance

	F (*P*-value)			
	
Characteristics	Men, N = 822		Women, N = 1,097	
		
	Metabolic syndrome	HOMA-IR ≥2.6†	Metabolic syndrome	HOMA-IR ≥2.6†
Age (years)	0.058 (0.810)	3.774 (0.052)	0.329 (0.566)	0.015 (0.902)
Smoking status, N (%)	2.097 (0.148)	4.359 (0.037)	1.252 (0.263)	3.667 (0.056)
Alcohol consumption, N (%)	6.959 (0.008)	10.29 (0.001)	3.454 (0.063)	0.306 (0.580)
Uric acid (mg/dL)	0.003 (0.954)	1.592 (0.207)	10.07 (0.002)	5.156 (0.023)
eGFR (mL/min/1.73 m^2^)	0.679 (0.410)	1.820 (0.178)	1.161 (0.282)	0.172 (0.679)
Tertile of hsCRP	1.165 (0.281)	0.177 (0.674)	0.098 (0.755)	1.394 (0.238)
Tertile of GGT	0.196 (0.658)	0.813 (0.368)	0.440 (0.507)	0.944 (0.331)
Tertile of hsCRP * Tertile of GGT	5.965 (0.015)	6.684 (0.010)	5.898 (0.015)	12.86 (<0.001)

Metabolic syndrome was defined as having three or more of the following conditions: obesity, raised blood pressure, hypertriglyceridemia, low HDL cholesterolemia, and impaired fasting blood glucose. † Insulin resistance was defined as HOMA-IR ≥2.6. The net effect of each interaction was estimated using a general linear model.

### Association between hsCRP and GGT levels, and metabolic syndrome and HOMA-IR, within selected subgroups

Next, to control potential confounding by MetS and insulin resistance, the data were further stratified by age, alcohol consumption, uric acid, HMW adiponectin, and medication (e.g., antihypertensive, antilipidemic, and diabetic medication) (Table 5). The ORs for MetS and HOMA-IR ≥2.6 increased significantly in relation to hsCRP and GGT in almost all the subgroups.

**Table 5:** Association between hsCRP and GGT levels, and metabolic syndrome and Insulin resistance, within selected subgroups

		**Metabolic syndrome OR(95% CI)**.						**HOMA-IR ≥2.6† OR(95% CI)**.					
			
		Tertile of hsCRP			Tertile of GGT			Tertile of hsCRP			Tertile of GGT		
					
		1st	2nd	3rd	1st	2^nd^	3rd	1st	2nd	3rd	1st	2nd	3rd
Characteristics		<0.360	0.360-0.800	>0.800 mg/L	<29	29-54	>54 IU/L	<0.280	0.280-0.650	>0.650 mg/L	<18	18-24	>24 IU/L
Age													
<65 years	650	1.00	2.13 (1.26-3.59)	3.76 (2.25-6.27)	1.00	2.66 (1.50-4.72)	3.55 (2.00-6.31)	1.00	2.50 (1.44-4.33)	4.50 (2.62-7.72)	1.00	2.47 (1.37-4.45)	3.04 (1.70-5.42)
≥65 years	969	1.00	1.21 (0.73-2.03)	1.88 (1.16-3.06)	1.00	1.84 (1.12-3.04)	3.80 (2.31-6.28)	1.00	2.16 (1.12-4.15)	3.87 (2.08-7.19)	1.00	2.16 (1.12-4.15)	3.87 (2.08-7.19)
Alcohol consumption													
Absent	829	1.00	1.42 (0.83-2.44)	2.27 (1.34-3.85)	1.00	1.94 (1.16-3.25)	3.04 (1.78-5.10)	1.00	1.11 (0.62-1.98)	2.23 (1.29-3.87)	1.00	4.72 (2.47-9.03)	7.41 (3.81-14.4)
Present	1090	1.00	1.74 (1.07-2.84)	2.91 (1.82-4.66)	1.00	2.65 (1.54-4.58)	4.89 (2.83-8.44)	1.00	4.50 (2.37-8.54)	7.30 (3.88-13.7)	1.00	1.62 (0.93-2.82)	2.66 (1.55-4.55)
Uric acid													
<5.0 (mean) mg/dL	978	1.00	1.51 (0.87-2.60)	3.21 (1.90-5.40)	1.00	2.39 (4.41-4.05)	3.53 (2.07-6.03)	1.00	1.98 (1.11-3.54)	3.57 (2.03-6.29)	1.00	2.79 (1.59-4.89)	3.46 (1.95-6.14)
≥5.0 (mean) mg/dL	941	1.00	1.68 (1.03-2.75)	2.14 (1.33-3.44)	1.00	2.24 (1.32-3.79)	4.04 (2.38-6.85)	1.00	1.68 (1.03-2.75)	2.14 (1.33-3.44)	1.00	2.24 (1.32-3.79)	4.04 (2.38-6.85)
HMW adiponectin													
<5.03 (median) μg/mL	963	1.00	1.64 (1.02-2.64)	2.74 (1.73-4.32)	1.00	2.63 (1.63-4.23)	3.52 (2.18-5.69)	1.00	2.63 (1.50-4.60)	4.57 (2.66-7.87)	1.00	1.86 (1.11-3.09)	3.23 (1.97-5.30)
≥5.03 (median) μg/mL	956	1.00	1.27 (0.71-2.27)	1.53 (0.85-2.75)	1.00	1.79 (0.96-3.34)	3.76 (2.04-6.96)	1.00	1.61 (0.84-3.10)	2.12 (1.11-4.05)	1.00	4.41 (2.07-9.42)	5.09 (2.33-11.1)
Medication‡													
Absent	1342	1.00	1.76 (1.09-2.85)	3.11 (1.95-4.93)	1.00	2.02 (1.23-3.31)	3.36 (2.06-5.50)	1.00	3.24 (1.87-5.62)	5.73 (3.34-9.84)	1.00	2.39 (1.43-3.97)	3.05 (1.84-5.07)
Present	577	1.00	1.18 (0.66-2.09)	1.87 (1.07-3.25)	1.00	2.22 (1.24-3.96)	3.55 (1.95-6.44)	1.00	1.12 (0.57-2.19)	2.07 (1.10-3.88)	1.00	2.94 (1.43-6.06)	6.09 (2.94-12.6)

Metabolic syndrome was defined as having three or more of the following conditions: obesity, raised blood pressure, hypertriglyceridemia, low HDL cholesterolemia, and impaired fasting blood glucose. † Insulin resistance was defined as HOMA-IR ≥2.6. ‡Medication was defined as antihypertensive, antilipidemic, or diabetic medication. Each tertile was calculated within sex and then combined to avoid the gender differences. Values adjusted for sex, age, smoking status, alcohol consumption, uric acid, and estimated glomerular filtration rate.

## Discussion

In 1,919 community-dwelling subjects, we determined the prevalence rate of MetS, as defined by modified NCEP-ATPIII criteria [20], and examined the association between hsCRP and GGT, and MetS and its components. MetS was common, occurring in 17.2% of men and 15.5% of women. In both men and women, the prevalence rate of MetS increased significantly in relation to hsCRP and GGT, even after adjusting for age, smoking status, drinking status, uric acid, and eGFR. The OR of MetS increased dose-dependently with increasing tertiles of hsCRP and GGT. In addition, we demonstrated that there is an interaction between increased hsCRP and GGT. The ORs of MetS and HOMA-IR ≥2.6 were significantly increased in relation to hsCRP and GGT in almost all the subgroups stratified by age, alcohol consumption, uric acid, HMW adiponectin, and medication. To our knowledge, this is the first study to indicate these associations of CRP and GGT with MetS and insulin resistance in about 2,000 community-dwelling subjects.

Systemic inflammation is closely associated with the pathogenesis of MetS. Several previous studies have demonstrated that elevated CRP was associated with increased odds of MetS after adjusting for potential confounding factors [23-26]. In a rural Chinese population, compared with subjects without components of MetS, those with 1, 2, 3, 4, and 5 components of MetS had ORs of 1.39, 1.08, 1.84, 2.65, and 1.21 for elevated CRP in men and 1.91, 2.06, 3.10, 4.06, and 6.01 in women, respectively [27].

In our study, higher GGT levels were also positively associated with MetS, independent of other confounders. Similar results have been reported in recent studies [28,29]. Nakanishi et al [28] demonstrated that serum GGT may be an important predictor for developing MetS in 2,957 metabolic syndrome-free men and 3,260 nondiabetic men aged 35-59 years. After adjustments for age, family history of diabetes, BMI, alcohol intake, smoking status, regular physical activity, and white blood cell count, increased serum GGT was related to the risk of developing MetS, even among individuals with normal GGT concentrations, this is a finding consistent with previous prospective reports looking at GGT. Among a total 3246 Korean adults, the number of MetS components, prevalence of MetS, and insulin resistance (HOMA-IR) increased closely as the quartile of serum GGT increased [30]. André et al also demonstrated that serum GGT is an important predictor for developing MetS in 1,659 men and 1,889 women without MetS at baseline [31]. Moreover, in a pooled logistic analysis after adjustments for age, alcohol intake, smoking status, physical activity, alanine aminotransferase, fasting insulin and HOMA-IR, high baseline GGT concentrations predicted future development of MetS defined by the IDF and AHA/NHLBI criteria after 4 y of follow-up [32]. In a community-based cohort study of 9,148 Korean adults that included 1056 men, the risk of MetS occurring increased across the baseline GGT quartiles, independent of age plus the time elapsed from visit 1 to visit 2, baseline MetS, uric acid, regular exercise, alcohol consumption, and smoking, and even after further updating GGT values during the follow-up [33]. We also suggested that higher serum GGT was significantly associated with MetS and its components in the same population and this association was related with insulin resistance, independent of other confounding factors [34]. A unique point of this result is that hsCRP and GGT were independently and synergistically associated with MetS, its components and insulin resistance.

The mechanisms by which hsCRP and GGT reflect the risk for MetS are not completely understood. However, systemic inflammation is closely involved in the pathogenesis of MetS, and thus, both elevated hsCRP and GGT may also reflect inflammation, which impairs insulin signaling in the liver, muscle, and adipose tissues [35]. Fat accumulation in the liver or adipose tissues can induce inflammatory cytokines such as tumor necrosis factor-α, interleukin-6, and interleukin-8 [36]. These cytokines produced by adipocytes stimulate the hepatic synthesis of CRP, which is an acute-phase protein, and influence insulin resistance, lipid and glucose metabolism. [37]. Moreover, high GGT is strongly associated with higher CRP levels [38], suggesting that this enzyme represents the expression of subclinical inflammation, and has a role in cellular stress [39]. On the other hand, we have been also reported that increased hsCRP and decreased high molecular weight (HMW) adiponectin are synergistically associated with the accumulation of metabolic disorders [40], however, both hsCRP and GGT were associated with insulin resistance also in subgroups stratified by HMW adiponectin. Furthermore, It has been demonstrated that increased GGT levels might be an antioxidant marker (defensive response) to oxidative stress or a direct marker of oxidative stress, which is involved directly in the generation of reactive oxygen species (ROS), especially in the presence of iron or other transition metals, inducing lipid peroxidation in human biological membranes [41,42].

There are some limitations to this study. First, our cross-sectional study design does not eliminate potential causal relationships between CRP, GGT and MetS. Second, the prevalence rate of MetS, GGT, and hsCRP categories is based on a single assessment of blood, which may introduce a misclassification bias. Third, we used BMI ≥25 to classify individuals with visceral obesity because waist circumference measurements were not available, which might have caused an under or over estimation of the effect of visceral obesity on MetS [43]. In fact, the prevalence rate of MetS in women was higher than those of the general reports in Japanese [1,44]. Fourth, serum GGT levels are various for same alcoholic consumption, and the possible association of the fatty liver with the presence of MetS, and with the elevated GGT could not be accurately explored. Thus, we demonstrated that GTT are independently associated with MetS after adjustment for alcoholic consumption and also in subgroup of drinker and non-drinker, suggesting drinking status does not dramatically affect the usefulness of γ-GTP as a biomarker for MetS risk. Fifth, the presence of viral hepatitis must be considered, but examinations of hepatitis B surface antigen and antibody to hepatitis C were not performed. In addition, the rate of taking antilipidemic medication in men with Mets was a little low; however we cannot explain that background. Therefore the demographics and referral source may limit generalizability.

## Conclusion

In conclusion, the present study showed that hsCRP and GGT levels are strongly associated with MetS or its components in the general population. The underlying mechanism behind this relationship is unclear, but seems to be independent of traditional cardiovascular risk factors such as age, smoking status, alcohol consumption, uric acid, or renal function. For community-dwelling healthy persons, prospective population-based studies are needed to investigate the mechanisms underlying this association to determine whether intervention, such as effective lifestyle modifications or medication (e.g., antihypertensive, antilipidemic, and diabetic medication) that decrease hsCRP and GGT in adults [45], will decrease the risk of MetS.

## Competing interests

The authors declare that they have no competing interests.

## Authors' contributions

RK, YT, and KK participated in the design of the study, performed the statistical analysis and drafted the manuscript. NO, TaK, and ToK contributed to acquisition of data and its interpretation. ST and MA contributed to conception and design of the statistical analysis. TM conceived of the study, participated in its design, coordination and helped to draft the manuscript. All authors read and approved the manuscript.
